# Cadherin‐6 controls neuronal migration during mouse neocortical development via an integrin‐mediated pathway

**DOI:** 10.1111/febs.70150

**Published:** 2025-05-28

**Authors:** Yuki Hirota, Rikaho Saito, Takao Honda, Hitomi Sano, Mayuko Hotta, Yukiko U. Inoue, Takayoshi Inoue, Kazunori Nakajima

**Affiliations:** ^1^ Department of Anatomy Keio University School of Medicine Tokyo Japan; ^2^ Laboratory of Molecular Biology, Department of Biofunctional Analysis Gifu Pharmaceutical University Japan; ^3^ Department of Information and Management Tokyo Online University Japan; ^4^ Department of Biochemistry and Cellular Biology National Institute of Neuroscience, National Center of Neurology and Psychiatry Tokyo Japan

**Keywords:** cadherin‐6, integrin, migration, neocortical development, neuron

## Abstract

During neocortical development, neuronal migration is highly regulated by multiple signaling cascades, including the cell adhesion molecules. Cadherin‐6 (CDH6), an unusual cadherin molecule containing an RGD integrin‐binding motif, has multiple functions in the developing nervous system, but whether it contributes to neuronal migration and positioning during neocortical development remains unknown. Here, we investigated the role of CDH6 in the developing cerebral cortex. *Cdh6* knockdown (KD) using *in utero* electroporation revealed that CDH6 inhibition caused impaired radial migration and abnormal positioning of neurons. Time‐lapse imaging analysis revealed that CDH6 is important for proper neuronal motility. Mechanistically, we show that CDH6 promotes the activation of integrin β1 on migrating neurons. The defect in neuronal migration caused by *Cdh6* KD was rescued by moderate overexpression of integrin β1 and a KD‐resistant form of wild‐type CDH6, but not by CDH6 with a mutated RGD motif. These results suggest that CDH6 is required for cortical excitatory neurons to migrate radially by controlling integrin‐mediated cell motility.

## Graphical abstract

Neuronal migration plays essential roles in the establishment of mammalian brains, and its impairment causes severe developmental abnormalities and behavioral and cognitive deficits. Here, we report that cadherin‐6 (CDH6), an unusual cadherin molecule containing an RGD integrin‐binding motif in the first extracellular domain, controls the motility of migrating neurons in the developing mouse cerebral neocortex by activating integrin β1 on migrating neurons.

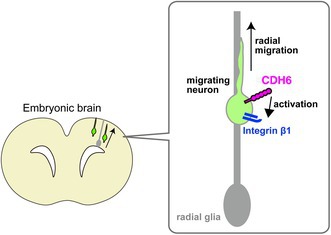

**Abbreviations**
CDH6: cadherin‐6
CP: cortical plate
FACS: fluorescence activated cell sorting
ICAM: intracellular adhesion molecule
IUE: *in utero* electroporation
IZ: intermediate zone
MAZ: multipolar cell accumulation zone
PLL: poly‐l‐Lysine
SLM: stratum lacunosum moleculare
VZ: ventricular zone

## Introduction

Neuronal migration plays essential roles in the establishment of the six‐layered laminar structure of the mammalian cerebral neocortex. During neocortical development, excitatory neurons generated in the ventricular zone (VZ) or subventricular zone migrate radially toward the pial surface to localize in the appropriate layer within the cortical plate (CP). During this process, neurons sequentially switch migration modes, including multipolar migration in the intermediate zone (IZ), radial glia‐guided locomotion in the IZ and CP, and terminal translocation in the superficial position of the CP [1, 2, 3, 4, 5]. These migration processes are the basis for proper neural circuitry and are highly controlled by various molecular mechanisms, including cell adhesion molecules.

Members of the cadherin superfamily, which act as mediators of cell adhesion through both homophilic and heterophilic binding in a calcium‐dependent manner, have multiple functions in the development, including cell sorting, tissue boundary formation, and cell migration. Among the members of the cadherin superfamily, Cadherin‐2 (CDH2) is known to have prominent and diverse roles during neocortical development, including maintenance of the VZ structure, correct polarization of newborn neurons, multipolar to bipolar transition, locomotion, and somal/terminal translocation [6]. On the contrary, although the expression of several cadherin members suggests their role in area boundary formation in the neocortex [7], whether cadherins other than CDH2 are also involved in neocortical development remains largely unknown.

Cadherin‐6 (CDH6) is a member of the classical type II cadherin family with abundant mRNA expression during neocortical development [7, 8, 9, 10]. Previous reports showed that CDH6 has functions in the developing nervous system, including neuronal targeting in the retina [11], forebrain–midbrain boundary formation [12], and determination of cortical neuron orientation [9, 13], but whether CDH6 contributes to neuronal migration and positioning during neocortical development remains unknown. In the present study, we investigated the expression pattern and function of CDH6 during neocortical development. shRNA‐mediated knockdown (KD) of *Cdh6* revealed that CDH6 plays an important role in the proper neuronal migration in both the neocortex and hippocampus, as well as in final positioning in the neocortex. Our results further suggest that CDH6 controls neuronal migration by regulating integrin activation.

## Results

### CDH6 protein is localized in the developing neocortex and hippocampus

**Fig. 1:**
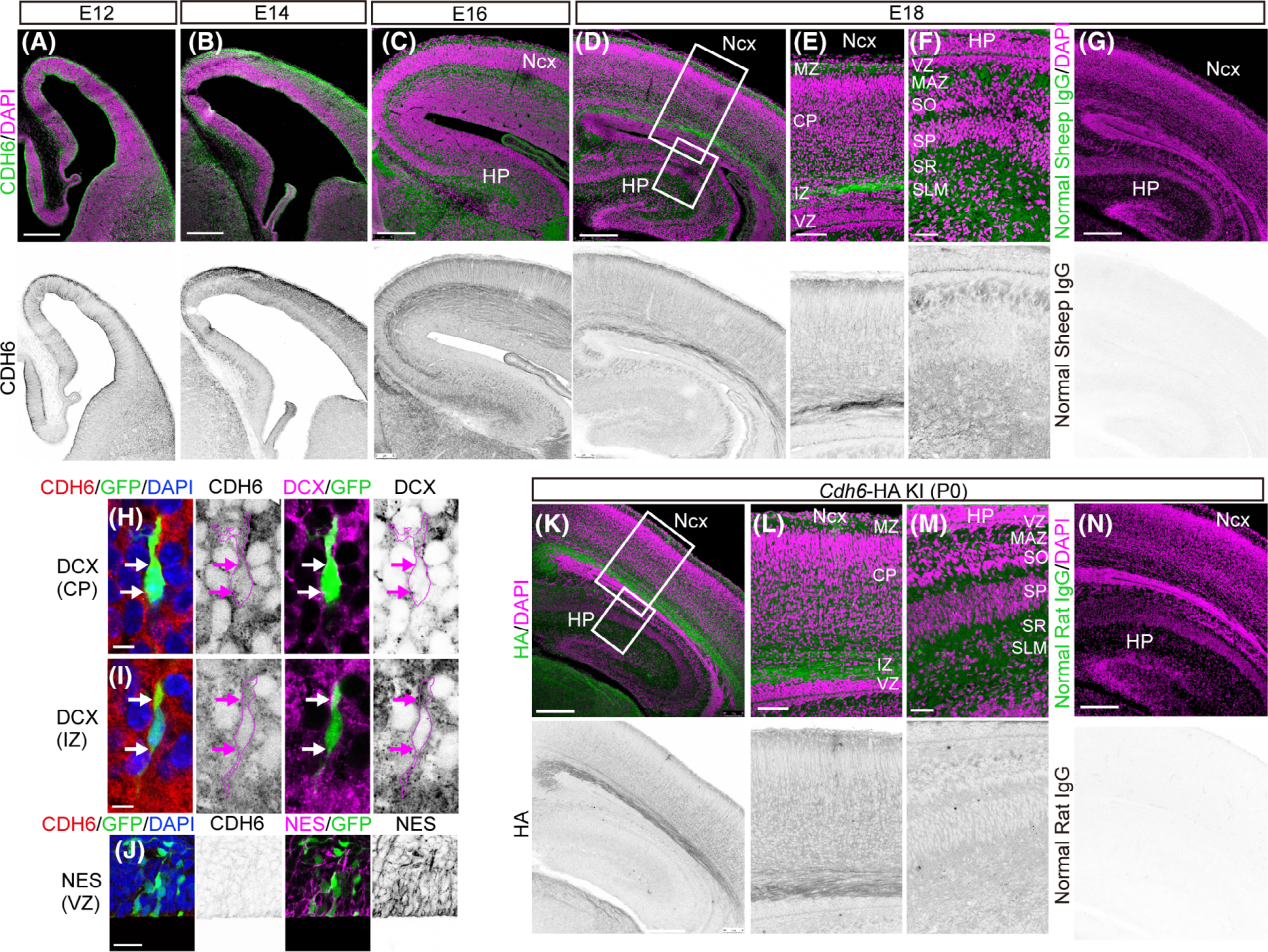
CDH6 protein is detected in the developing mouse cerebral cortex. (A–G) Immunostaining for CDH6 (green) and DAPI (magenta) using sections from the cerebral cortex at E12.0 (A) (*n* = 6), E14.0 (B) (*n* = 6), E16.0 (C) (*n* = 4) and E18.0 (D–F) (*n* = 10) of the ICR mouse. The boxed regions in (D) are shown at higher magnification in (E) and (F). (G) Control staining with normal sheep IgG instead of CDH6 antibody (*n* = 10). The lower panels in (A–G) show grayscale, single‐channel images of CDH6 (A–F) and normal sheep IgG (G), respectively. (H–J) The cerebral cortices (E17.0) that had been electroporated with CAG‐EGFP plasmid on E14.0 were stained for DCX (H, I) (*n* = 4) and Nestin (NES) (*n* = 4) (J). Arrows in (H) and (I) indicate colocalization of immunosignals of CDH6/DCX in EGFP‐positive neurons in CP and IZ, respectively. Cell outline is highlighted using the GFP signal as a guide in the CDH6 and DCX panels (magenta lines in H and I). The second panels from the left in (H–J) show CDH6 grayscale, single‐channel images. The fourth panels from the left in (H–I) show DCX grayscale, single‐channel images. The fourth panel from the left in (J) shows a NES grayscale, single‐channel image. (K–M) Immunostaining for HA (green) and DAPI (magenta) using sections from the cerebral cortex at P0 of the *Cdh6*‐HA KI mouse (*n* = 4). Boxed regions in (K) are shown at higher magnification in (L) and (M). (N) Control staining with normal rat IgG instead of HA antibody (*n* = 4). The lower panels in (K–N) show grayscale, single‐channel images of CDH6 (K–M) and normal rat IgG (N), respectively. Scale bars: 100 μm in (A, B, C, D, G, K, N); 40 μm in (E, F, L, M); 5 μm in (H, I); 20 μm in (J). CP, cortical plate; DCX, Doublecortin; HP, hippocampus; IZ, intermediate zone; MAZ, multipolar cell accumulation zone; MZ, marginal zone; Ncx, neocortex; NES, Nestin; SLM, stratum lacunosum moleculare; SO, stratum oriens; SP, stratum pyramidale; SR, stratum radiatum; VZ, ventricular zone.

Although previous papers showed that *Cdh6* mRNA is expressed in the developing neocortex [7, 8, 9, 10], its protein localization and function during cortical development remain largely unknown. Immunostaining of coronal sections of mouse embryonic brain with an antibody against CDH6 (Fig. 1A–G) revealed that CDH6 protein was widely detected in the cortical wall at embryonic day (E) 12, E14, and E16 (Fig. 1A–C). At E18.0, CDH6 protein was distributed in the IZ and CP, with a strong expression in the marginal zone and little expression in the VZ (Fig. 1D,E). In the IZ, strong protein signals were also observed in axons (Fig. 1E). In addition, CDH6 was also detected in the developing hippocampus (Fig. 1D,F) mainly from the multipolar cell accumulation zone (MAZ) to the stratum lacunosum moleculare (SLM) (Fig. 1F), including where migrating neurons are present [14], with weak signals in the VZ (Fig. 1C,D,F). We also examined CDH6 expression in migrating neurons and at the apical side of the VZ using E17 neocortex transfected at E14 with a GFP‐expressing vector by *in utero* electroporation (IUE) combined with staining with antibodies for Doublecortin (DCX) (newborn neuron marker) and Nestin (radial glia marker). CDH6 was clearly detected in DCX‐positive migrating neurons (Fig. 1H,I), whereas it was only weakly expressed in Nestin‐positive radial glia at the apical side of the VZ (Fig. 1J). To further confirm the specificity of the CDH6 protein signals in the developing neocortex and hippocampus, we generated a *Cdh6‐HA* tag knock‐in mouse line. Immunostaining with an antibody against HA showed almost the same localization pattern as observed by CDH6 immunostaining in both neocortex and hippocampus (Fig. 1K–N). Although we cannot exclude the possibility of cross‐reactivity of the anti‐CDH6 antibody with other cadherins, the similarity of the localization pattern between CDH6 protein signals in wild‐type mice and HA signals in *Cdh6‐HA* tag knock‐in mice supports that the CDH6 immunostaining is specific for CDH6. These results suggest a possible role for CDH6 in neuronal migration in the developing neocortex and hippocampus.

### CDH6 cell‐autonomously controls neuronal migration and positioning in the developing neocortex

**Fig. 2:**
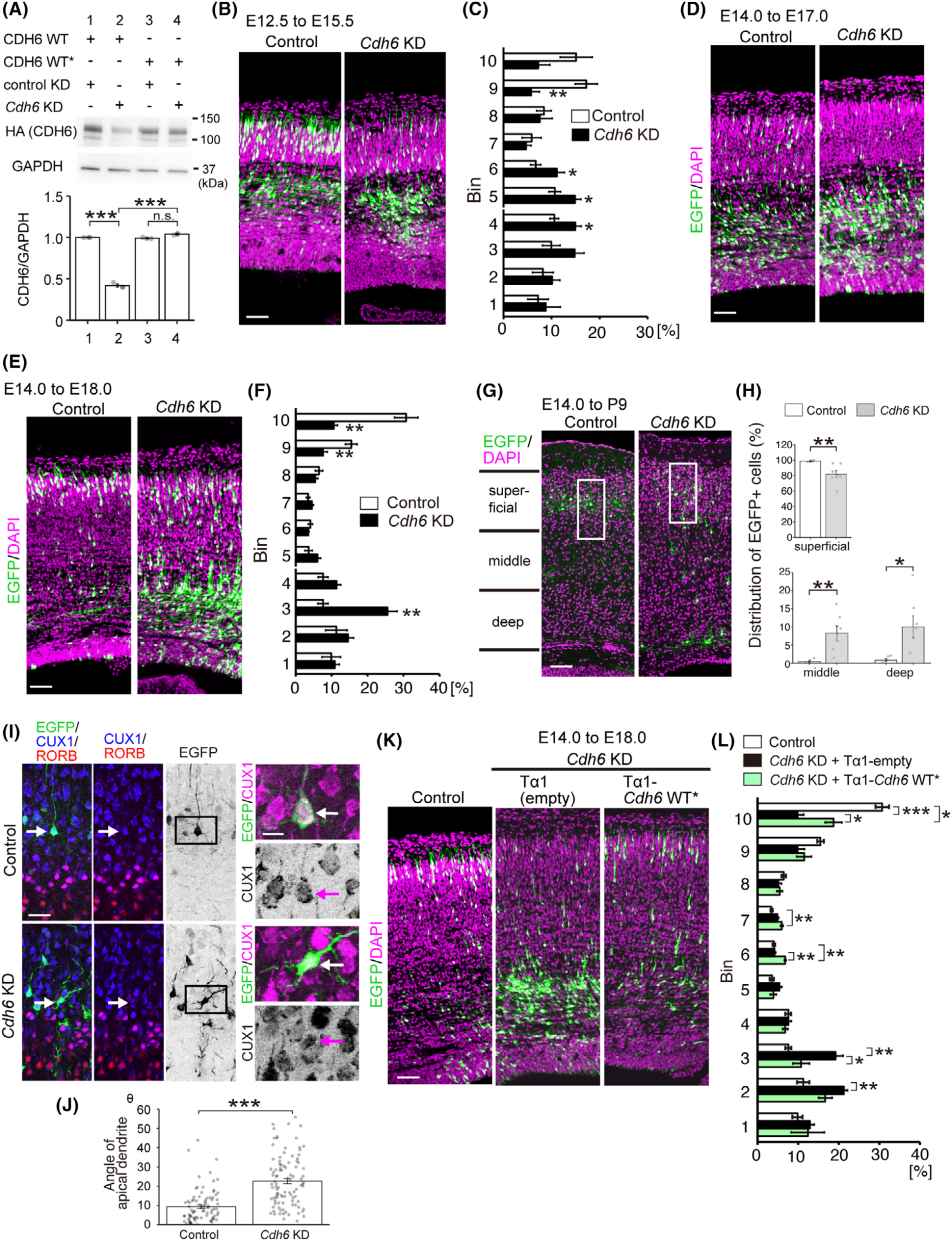
CDH6 controls neuronal migration in the developing neocortex. (A) Knockdown verification. HEK293T cells were co‐transfected with vectors expressing HA‐tagged *Cdh6* WT or *Cdh6* WT*, a KD‐resistant form of *Cdh6*, with control or *Cdh6* KD vectors, and the cell extracts were subjected to western blotting analysis with antibodies against HA or GAPDH (a loading control). The graph represents the normalized CDH6/GAPDH ratio (*n* = 4) (1 vs. 2, ***P = 0.0003; 2 vs. 4, ***P = 0.0002) (one‐way ANOVA with Tukey's *post hoc* test). Data are mean ± SEM. (B, C) E12.5 embryos were electroporated with the control or *Cdh6* KD vector and CAG‐EGFP, and the distribution of EGFP‐labeled cells was examined at E15.5. Graphs show quantification of cell migration (control, *n* = 5; *Cdh6* KD, *n* = 5). Data are mean ± SEM. Statistically significant differences were observed in bin 4 (*P = 0.023), bin 5 (*P = 0.042), bin 6 (*P = 0.047), and bin 9 (**P = 0.004) (one‐way ANOVA with Tukey's *post hoc* test). (D, E) E14.0 embryos were electroporated with the control or *Cdh6* KD vector and CAG‐EGFP, and the distribution of EGFP‐labeled cells was examined at E17.0 (D) (*n* = 4) and E18.0 (E) (*n* = 4). (F) Graphs show quantification of cell migration analyzed at E18.0 (control, *n* = 4; *Cdh6* KD, *n* = 4). Data are mean ± SEM. Statistically significant differences were observed in bin 3 (**P = 0.0010), bin 9 (**P = 0.0079), and bin 10 (**P = 0.0011) (Student's *t*‐test). (G) E14.0 embryos were electroporated with the control or *Cdh6* KD vector and CAG‐EGFP, and the distribution of EGFP‐labeled cells was examined at P9 (control, *n* = 6; *Cdh6* KD, *n* = 7). The boxed regions are shown at higher magnification in (I). (H) Distribution of EGFP‐labeled cells in the cortical walls at P9. The percentage of EGFP‐labeled cells in each group is significantly different between control and *Cdh6* KD (control, *n* = 6; *Cdh6* KD, *n* = 7, Student's *t*‐test, superficial, P** = 0.0071; middle, P** = 0.0034; deep, P* = 0.0194). Data are mean ± SEM. (I) Morphology of EGFP‐labeled CUX2 (blue)‐positive RORB (red)‐negative neurons (indicated by arrows) located in the layer II/III at P9 (left). The boxed regions in left panels are shown at higher magnification in right panels, in which colocalization of EGFP (green) and CUX1 (magenta) is shown. (J) The angle of the apical dendrite was significantly larger in the *Cdh6* KD case compared with the control case (control, 10.3 ± 1.3, *n* = 88 cells from 6 mice; *Cdh6* KD, 35.7 ± 3.1, *n* = 120 cells from 6 mice; ***P < 0.001) (Student's *t*‐test). Data are mean ± SEM. (K) E14.0 embryos were electroporated with the Tα1 empty vector or Tα1‐*Cdh6* WT* together with *Cdh6* KD vector and CAG‐EGFP, and the distribution of EGFP‐labeled cells was examined at E18.0. (L) Graphs show the quantification of cell migration analyzed at E18.0 (Control, *n* = 4; *Cdh6* KD + Tα1 empty, *n* = 6; *Cdh6* KD + Tα1‐*Cdh6* WT*, *n* = 4). Data are mean ± SEM. Statistically significant differences were observed in bin 2 (Control vs. *Cdh6* KD + Tα1 empty, **P = 0.006), bin 3 (Control vs. *Cdh6* KD + Tα1 empty, **P = 0.002; *Cdh6* KD + Tα1 empty vs. *Cdh6* KD + Tα1‐*Cdh6* WT*, *P = 0.018), bin 6 (Control vs. *Cdh6* KD + Tα1‐*Cdh6* WT*, **P = 0.002; *Cdh6* KD + Tα1 empty vs. *Cdh6* KD + Tα1‐*Cdh6* WT*, **P = 0.002), bin 7 (Control vs. *Cdh6* KD + Tα1‐*Cdh6* WT*, **P = 0.004), and bin 10 (Control vs. *Cdh6* KD + Tα1 empty, ***P < 0.001; *Cdh6* KD + Tα1 empty vs. *Cdh6* KD + Tα1‐*Cdh6* WT*, *P = 0.033; Control vs. *Cdh6* KD + Tα1‐*Cdh6* WT*, *P = 0.011) (one‐way ANOVA with Tukey's *post hoc* test). Each section was stained with DAPI (magenta). Scale bars: 50 μm in B, D, E, G, K; 30 μm in left panel in I; 10 μm in right panel in (I). KD, knockdown; WT, wild‐type.

We then investigated the function of CDH6 during neocortical development using shRNA‐mediated KD. We generated a *Cdh6* KD vector that significantly reduced the protein level of exogenously introduced CDH6 in HEK293T cells (Fig. 2A). In addition, we generated an shRNA‐resistant form of CDH6 (Cdh6 WT*), and confirmed its resistance to the *Cdh6* KD suppression in HEK293T cells (Fig. 2A). To investigate the effect of CDH6 inhibition on neurons generated in the early stages of development, *Cdh6* KD or control vector was introduced with an EGFP‐expression vector into the VZ of the cerebral cortex at E12.5 by IUE. When analyzed at E15.5, the majority of labeled control cells had entered the CP. In contrast, a significantly higher number of labeled cells in the *Cdh6* KD brains remained in the IZ (Fig. 2B,C). These results suggest that CDH6 is required for the migration of early‐born neurons. We also examined the effect of CDH6 inhibition on late‐born neurons by IUE at E14.0, followed by examination on Day 3 after the IUE. In the control case, a considerable number of EGFP+ cells had already entered the CP, whereas in the *Cdh6* KD case, most of the labeled cells remained in the IZ (Fig. 2D). The distribution of EGFP‐labeled cells was clearly shifted to a deeper position than in the control when examined 4 days after IUE (Fig. 2E,F). When examined at P9, a significant number of *Cdh6* KD neurons were still located in the middle and deep parts of the cortex, whereas almost all control neurons settled in the superficial part of the cortical wall (Fig. 2G,H), suggesting that inhibition of CDH6 disrupted normal neuronal migration, rather than transiently delaying migration. To compare neuronal morphology of superficial layer neurons, we costained the sections for RORB and CUX1, which are markers for layer IV and superficial layer neurons, respectively [15, 16, 17], to confirm that the cells we analyzed were comparable in terms of their neuronal subtype, considering that the microenvironment in which the neurons are positioned could affect the final specification/differentiation of cortical neurons [18, 19, 20]. In layers II/III, some *Cdh6* KD neurons that were positive for CUX1 and negative for RORB displayed a tilted morphology with respect to the pial surface compared with CUX1‐positive/RORB‐negative control neurons (insets in Fig. 2G,I,J), consistent with the previously reported requirement for CDH6 in establishing the postmigratory orientation of neurons [13]. Coexpression of CDH6 WT* using the neuron‐specific Tα1 promoter with a *Cdh6* KD vector significantly rescued the migration defect caused by the *Cdh6* KD (Fig. 2K,L), confirming the specificity of the KD effect. Some GFP‐positive neurons were distributed in the deep part of the cortex, probably due to differences in the efficiency of electroporation between cells (Fig. 2K,L). These results indicate that CDH6 is required for neurons to migrate radially toward the pial surface in a cell‐autonomous manner.

### CDH6 is involved in the proper cell motility of neurons

**Fig. 3:**
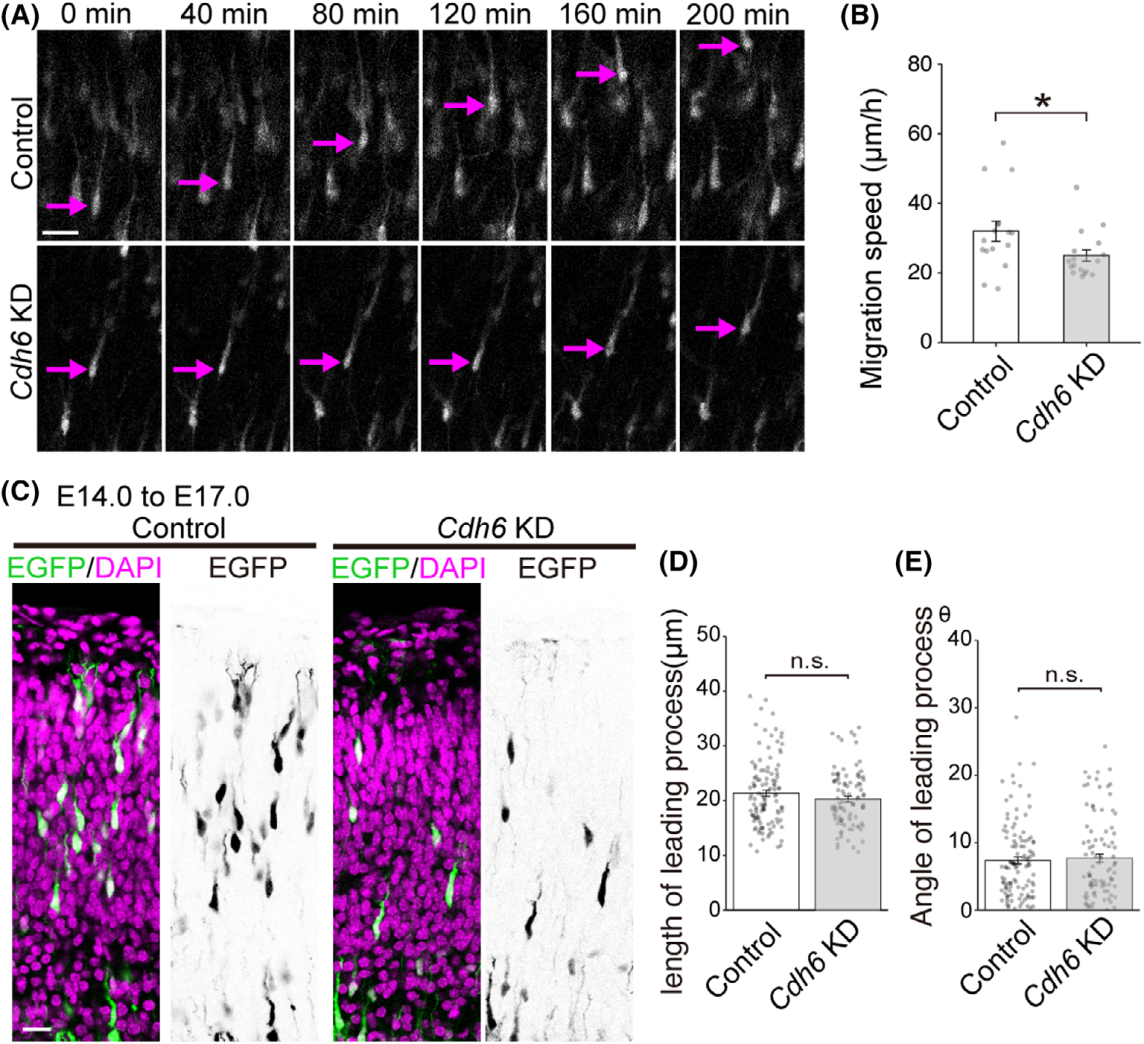
CDH6 is involved in the proper neuronal motility. (A) Time‐lapse images of control and *Cdh6* KD neurons migrating in the IZ, taken at 10‐min intervals. Images taken every 40 min are shown. Arrows indicate representative tracked neurons. (B) Migration speed was slower in the *Cdh6* KD case than in the control case (control, 32.0 ± 2.9 μm·h^−1^, *n* = 16 cells from 3 mice; *Cdh6* KD, 25.1 ± 1.7 μm·h^−1^, *n* = 18 cells from 3 mice; P* = 0.0361) (Student's *t*‐test). Data are mean ± SEM. (C) E14.0 embryos were electroporated with the control or *Cdh6* KD vector and CAG‐EGFP, and the length and orientation of leading processes of EGFP‐labeled neurons migrating in the superficial part of the IZ and CP were examined at E17.0. (D) The length of leading process was not altered in the *Cdh6* KD case compared with the control case (control, 21.3 ± 0.6 μm, *n* = 114 cells from 4 mice; *Cdh6* KD, 20.3 ± 0.5 μm, *n* = 96 cells from 4 mice; *P* = 0.17) (Student's *t*‐test). Data are mean ± SEM. (E) The angle of the leading process was not changed in the *Cdh6* KD case compared with the control case (control, 7.4 ± 0.7 μm·h^−1^, *n* = 114 cells from 4 mice; *Cdh6* KD, 7.7 ± 0.6, *n* = 96 cells from 4 mice; *P* = 0.67) (Student's *t*‐test). Data are mean ± SEM. Scale bars: 50 μm in (A), 30 μm in (C). CP, cortical plate; IZ, intermediate zone; KD, knockdown.

Next, we examined the effect of CDH6 inhibition on the motility of radially migrating cortical neurons using time‐lapse imaging. *Cdh6* KD or control vector was introduced with an EGFP‐expressing vector at E14.0 by IUE, and time‐lapse imaging was performed 3 days after the IUE. At the beginning of the live imaging, many EGFP‐labeled neurons were located in the deep part of the IZ. In both control and *Cdh6* KD cases, many EGFP‐labeled neurons radially migrated in the IZ and CP toward the pial surface during the recording period. Quantitative analysis of the migration speed in the CP revealed that the *Cdh6* KD neurons migrated significantly slower than the control cells (Fig. 3A,B). We also examined the length and orientation of the leading processes of migrating neurons using brain sections obtained from E17.0 neocortex electroporated at E14.0. As a result, there was no significant difference (Fig. 3C–E), suggesting that CDH6 is required for the motility rather than the morphology of the leading processes of migrating neurons. These results suggest that CDH6 facilitates cell motility during neuronal migration.

### CDH6 controls neuronal migration in an RGD‐dependent manner by activating integrin β1

**Fig. 4:**
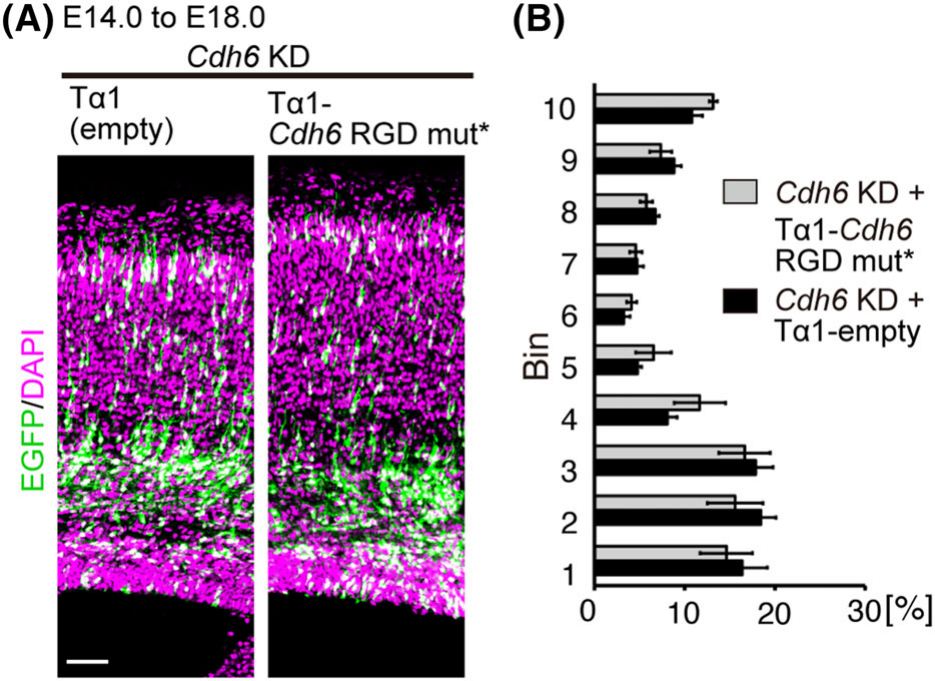
CDH6 regulates neuronal migration in the developing cortex in an RGD motif‐dependent manner. (A) E14.0 embryos were electroporated with Tα1 empty vector or Tα1‐*Cdh6* RGD mut* with *Cdh6* KD vector and CAG‐EGFP, and the distribution of EGFP‐labeled cells was examined at E18.0. (B) Graphs show the quantification of cell migration in (A) (Tα1 empty, *n* = 6; Tα1‐*Cdh6* RGD mut*, *n* = 5). Data are mean ± SEM. No significant difference was observed (Student's *t*‐test). Sections in (A) were stained with DAPI (magenta). Scale bar: 50 μm in (A). KD, knockdown; mut, mutant.

Next, we investigated how CDH6 controls neuronal migration. CDH6 is a cadherin that binds to integrins through an arginine‐glycine‐aspartic acid (RGD) motif in the first extracellular domain. In cancer cells and platelets, CDH6 binds to integrin through this motif to promote cell adhesion, migration, and proliferation [21, 22]. In addition, a recent paper showed that CDH6 controls the orientation of postmigratory cortical neurons, possibly through interaction with integrin [13]. We thus asked whether CDH6 also regulates radial migration by binding to integrin. To address this question, we generated an expression vector of CDH6 with a mutated RGD motif by changing the sequence of RGD to RAE (CDH6 RGD mut). Equivalent mutations in CDH6 and other RGD‐containing cadherins (including CDH17 and CDH5) have been shown to specifically disrupt association with integrins, but not homophilic interactions [23]. Expression of an shRNA‐resistant CDH6 RGD mutant (CDH6 RGD mut*) failed to rescue the impaired radial migration caused by the *Cdh6* KD (Fig. 4A,B), suggesting that CDH6 controls radial neuronal migration via the RGD motif, possibly by binding to integrins.

**Fig. 5:**
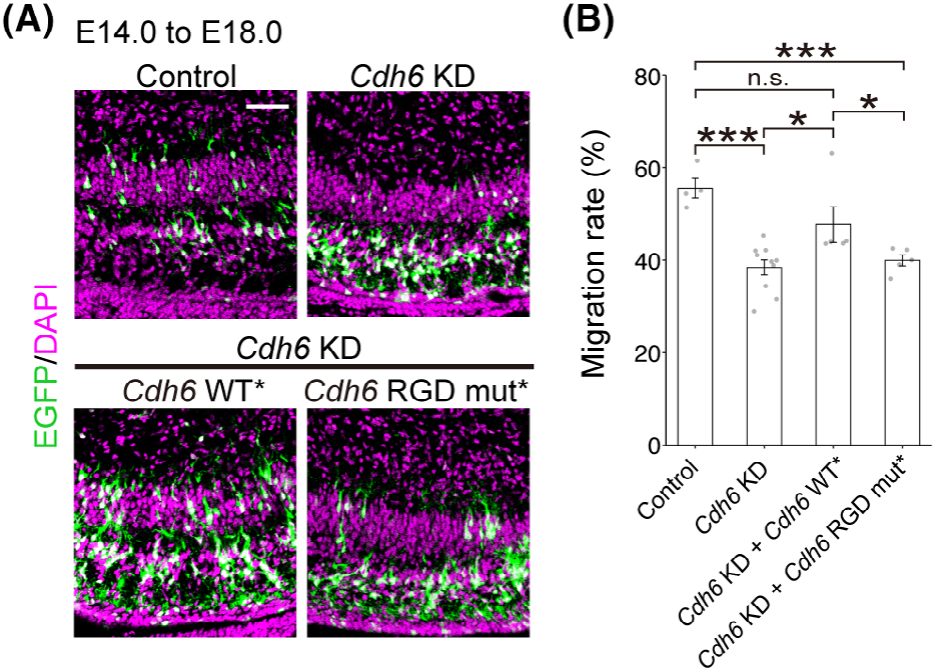
CDH6 regulates neuronal migration in the hippocampal CA1 in an RGD motif‐dependent manner. (A) The CA1 regions that had been electroporated with control or *Cdh6* KD vector with CAG‐EGFP plasmid at E14.0 were analyzed at E18.0. (B) Graphs show quantification of cell migration in (A) (control, *n* = 4; *Cdh6* KD, *n* = 10; *Cdh6* KD + *Cdh6* WT*, *n* = 5; *Cdh6* KD + *Cdh6* RGD mut*, *n* = 5). Data are mean ± SEM. Statistically significant differences were observed in control vs. *Cdh6* KD (***P < 0.001), control vs. *Cdh6* KD + *Cdh6* RGD mut* (***P < 0.001), *Cdh6* KD vs. *Cdh6* KD + *Cdh6* WT* (*P = 0.025), and *Cdh6* KD + *Cdh6* WT* vs. *Cdh6* KD + *Cdh6* RGD mut* (*P = 0.044) (Tukey–Kramer test). Scale bar: 50 μm in (A). KD, knockdown; mut, mutant; WT, wild‐type.

The distribution of CDH6 in the developing hippocampus (Fig. 1F,M) suggests the possibility that CDH6 also controls neuronal migration in the hippocampus. Thus, we next examined whether CDH6 inhibition would affect neuronal migration in the hippocampal CA1 region, where the neuronal migration profile was previously well described [14, 24]. *Cdh6* KD or control vectors were introduced into the VZ in CA1 using IUE at E14.0. As a result, the distribution of the EGFP‐labeled cells was shifted to a deeper position in the *Cdh6* KD 4 days after the IUE (Fig. 5A,B), indicating that CDH6 is required for normal neuronal migration in this region. Similar to the results in the neocortex, the migration defect caused by *Cdh6* KD was significantly restored by the coexpression of CDH6 WT*, but not CDH6 RGD mut* (Fig. 5A,B). These results suggest that CDH6‐mediated regulation of integrins to support neuronal migration is shared between the neocortex and hippocampus.

**Fig. 6:**
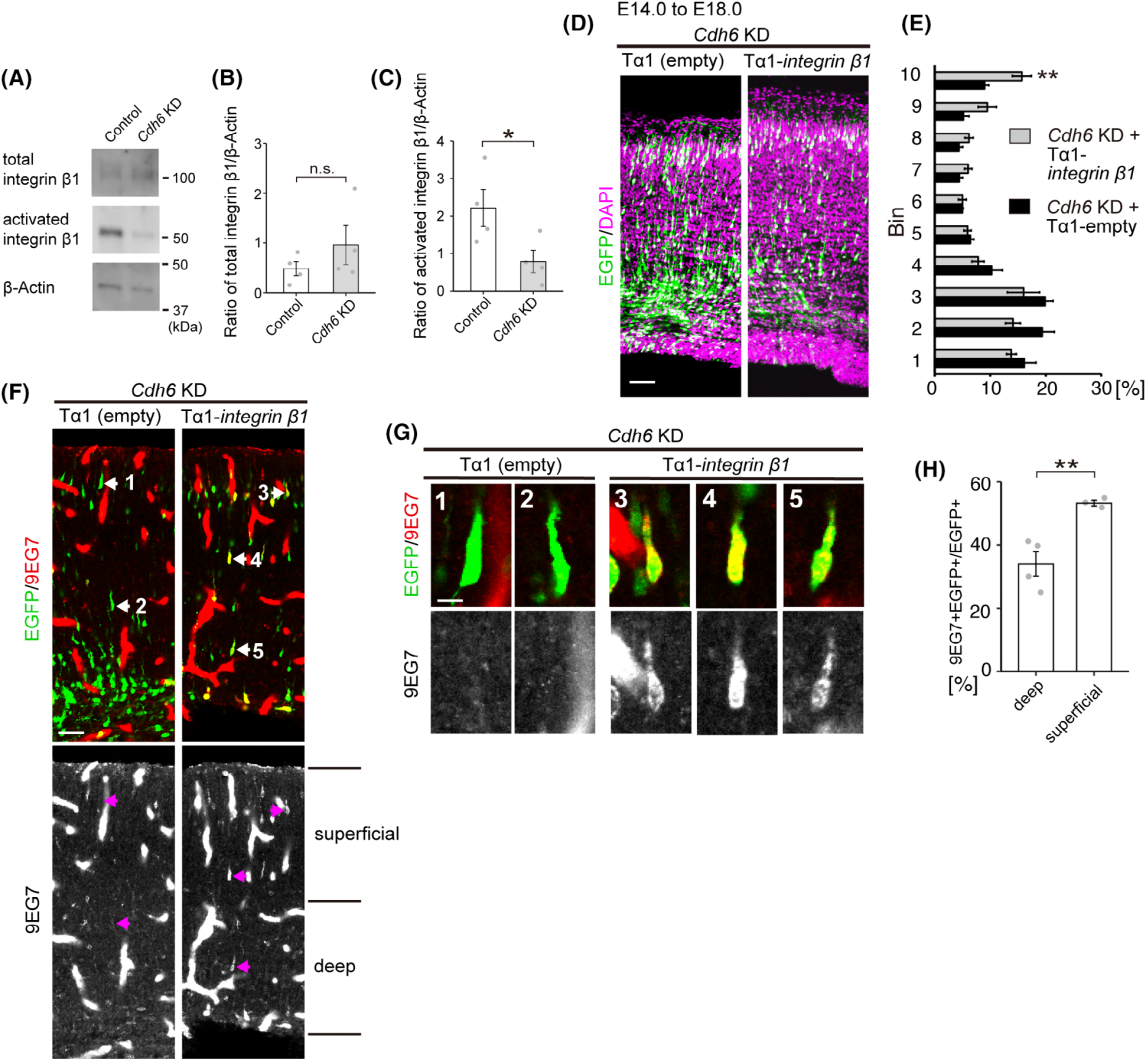
CDH6 regulates neuronal migration in the developing cortex through activation of integrin β1. (A) *In vitro* integrin activation assay. Control or *Cdh6* KD vectors together with CAG‐EGFP were introduced into E14.0 cortex by IUE. Three days after IUE, GFP‐labeled cells were collected by FACS, and then plated on PLL‐coated dishes. The amount of activated integrin β1 was quantified by the amount of bound 9EG7 antibody. (B, C) Normalized graphs of total integrin β1/β‐Actin ratio (B) and activated integrin β1/β‐Actin ratio (C), respectively (*n* = 4). Each bar represents the mean relative intensity ± SEM of the signal. *P = 0.048 (Student's *t*‐test). (D) E14.0 embryos were electroporated with Tα1 empty vector or Tα1‐*integrin β1* with *Cdh6* KD and CAG‐EGFP, and the distribution of EGFP‐labeled cells was examined at E18.0. (E) Graphs show the quantification of cell migration in (D) (Tα1 empty vector, n = 6; Tα1‐*integrin β1*, *n* = 7). Statistically significant differences were observed in bin 10 (**P = 0.006) (Student's *t*‐test). Data are mean ± SEM. (F) Immunostaining with 9EG7 (red) and for EGFP (green) using sections from the E18.0 cerebral cortex, which were electroporated with Tα1 empty vector (left) or Tα1‐*integrin β1* (right) with *Cdh6* KD and CAG‐EGFP vectors at E14.0. 9EG7 signal was detected in the EGFP‐positive neurons of Tα1‐*integrin β1*‐electroporated samples (arrows in right panels), but not in those of Tα1 empty vector‐electroporated samples (arrows in left panels) (*n* = 4). (G) High magnification images of EGFP‐positive neurons pointed by arrows in (F) (*n* = 4). (H) Percentage of 9EG7‐positive cells out of total GFP cells in the superficial and deep halves of the cortical wall of E18.0 cerebral cortex, which were electroporated with Tα1‐*integrin β1* with *Cdh6* KD and CAG‐EGFP vectors at E14.0. Data are mean ± SEM. Statistical analyses were performed using Student's *t*‐test (**P = 0.014, *n* = 3). Sections in (D) were stained with DAPI (magenta). Scale bars: 50 μm in (D, F); 10 μm in (G). FACS, fluorescence activated cell sorting; IUE, *in utero* electroporation; KD, knockdown; PLL, poly‐l‐Lysine.

Next, we tested whether CDH6 is involved in the integrin activation. Integrin β1 is one of the most highly expressed integrins in the developing neocortex [25]. Although the potential role of integrin β1 in the radial neuronal migration during development has long been controversial [25, 26, 27, 28, 29, 30], a recent paper demonstrated a cell‐autonomous requirement for integrin β1 in migrating cortical neurons [31]. Thus, we focused on integrin β1 and tested whether inhibition of CDH6 would affect integrin β1 activation using the *in vitro* integrin activation assay. *Cdh6* shRNA vectors or control vectors together with an EGFP‐expressing vector were introduced into the E14.0 cortex by IUE. Three days after IUE, when many transfected neurons were migrating along the radial glial fibers, EGFP‐labeled neurons were collected by fluorescence activated cell sorting (FACS), plated on poly‐l‐Lysine (PLL)‐coated dishes, and then incubated with an integrin β1 active conformation‐specific antibody, 9EG7 [32, 33]. Results showed that *Cdh6* KD significantly decreased the amount of 9EG7 antibody bound to the collected cells without affecting the total amount of integrin β1 (Fig. 6A–C), suggesting that CDH6 is required for proper activation of integrin β1 in migrating neurons. Consistent with this, coexpression of integrin β1 under the Tα1 promoter partially rescued the migration defects caused by the *Cdh6* KD (Fig. 6D,E). When the sections were stained with 9EG7 (Fig. 6F,G), we found that, after integrin β1 overexpression, *Cdh6* KD cells that were rescued in their migration to the superficial half of the cortical wall showed a higher proportion of 9EG7‐positive cells than those located in the deep half, suggesting that the increased amount of active integrin β1 in migrating neurons allowed them to reach the superficial side (Fig. 6H). The 9EG7‐positive signals were barely detectable in control cells using this method (left panels in Fig. 6F and left panels in Fig. 6G), suggesting that at least some of the overexpressed integrin β1 is activated in the integrin β1‐overexpressing neurons. Taken together, these results suggest that CDH6 cell‐autonomously activates integrin β1 in neurons, which is required for the proper neuronal migration in the developing neocortex.

## Discussion

Precise regulation of neuronal migration and positioning is important for the formation of the elaborate laminar structure of the neocortex. In the present study, we demonstrate that CDH6 plays an essential role in neuronal migration and positioning of excitatory neurons in the developing neocortex. Cortical neurons use radial glial fibers as a scaffold for migration in the IZ and CP. Since CDH6 protein and *Cdh6* mRNA are slightly expressed in the radial glia in the VZ in addition to migrating neurons (Fig. 1E,J) [7, 8, 10], we cannot exclude the possibility that reduced CDH6 protein expression in the radial glia also affects neuronal migration in the *Cdh6* KD experiments. However, our rescue experiment, in which co‐expression of a KD‐resistant form of CDH6 under the neuron‐specific promoter rescued the migration defects caused by *Cdh6* KD, clearly indicates the requirement of CDH6 in migrating neurons.

Our rescue experiments using CDH6 with a mutated RGD motif revealed that CDH6 function in neuronal migration depends on the RGD motif. Emerging evidence suggests that CDH6 exerts diverse functions via the RGD motif. Integrin members generally recognize RGD ligand proteins to promote cell motility [34]. Particularly, RGD cadherins and integrins play a major role in the metastatic progression of cancer. In ovarian and renal cancer metastasis, upregulated CDH6 activates integrin αIIbβ3, which in turn activates integrin α2β1 to promote tumor motility and migration [22]. Other RGD cadherins, CDH17 and CDH5, have been implicated in promoting liver and lung metastasis by interacting with the a2β1 integrin via the RGD motif [35]. Several signaling pathways are known to be commonly involved in neuronal and cancer cell migration. For example, SLIT/ROBO signaling, which regulates axon guidance and neuronal migration, is increased or decreased in pancreatic cancer [36, 37]. Trio, a member of the Rho guanine nucleotide exchange factors involved in the migratory behavior of various cancer cells, controls the radial migration of cortical projection neurons [38]. Thus, CDH6 may represent a novel common mechanism shared by cortical neurons and cancer cells for cell migration. The CDH6 RGD also plays physiological roles in developmental and homeostatic contexts. In thrombus formation, CDH6 promotes platelet adhesion by binding to the αIIβb3 integrin with the RGD motif [21]. In the developing neocortex, CDH6 protein overexpression affects the orientation of postmigratory neurons in an RGD motif‐dependent manner [13]. Taken together, our results reveal a novel physiological function for CDH6 via the RGD motif in regulating neuronal migration during neocortical development.

**Fig. 7:**
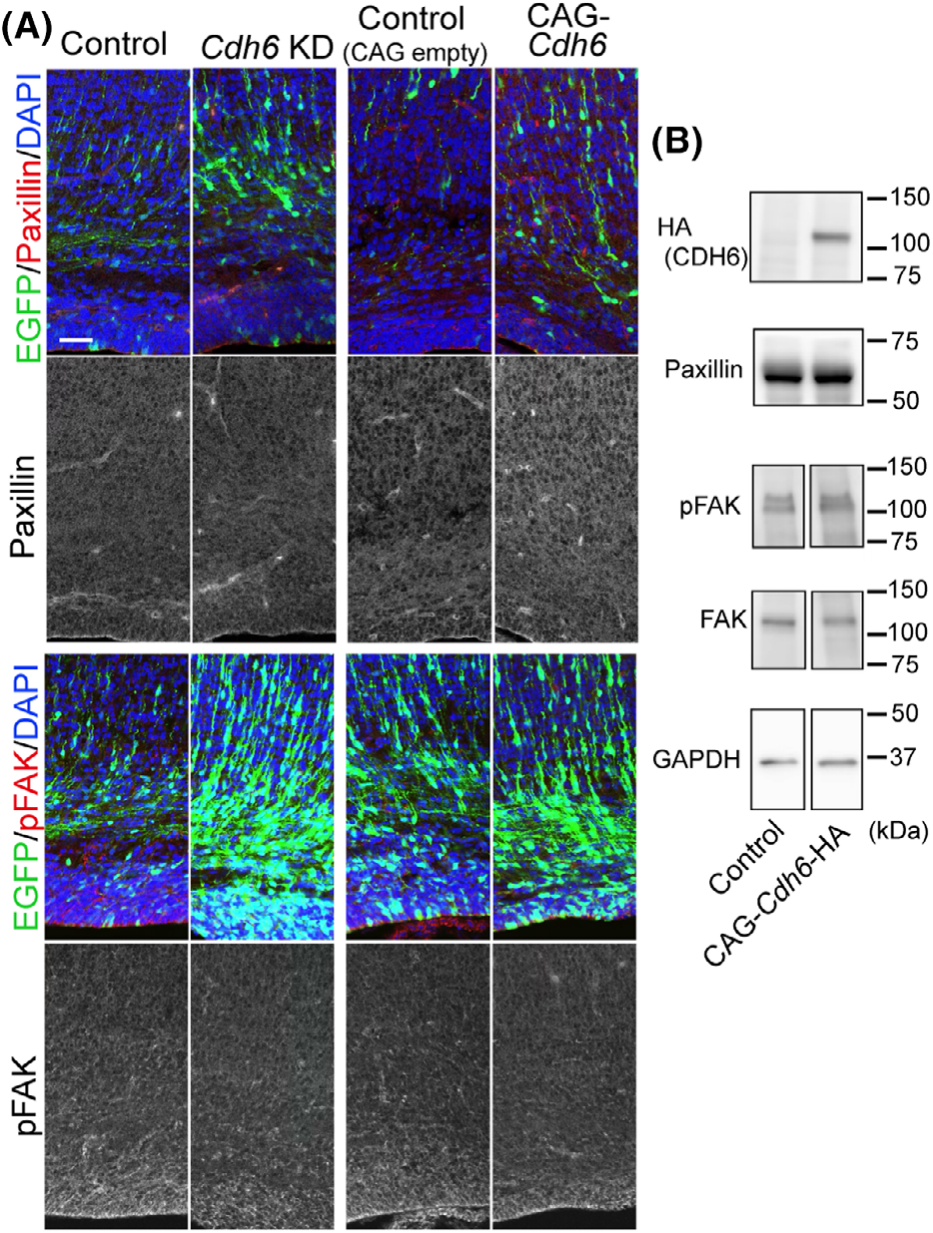
KD and overexpression of *Cdh6* did not alter the levels of Paxillin protein and phosphorylated AKT. (A) Immunostaining for Paxillin (top, red) or pFAK (red, bottom) and EGFP (green) using sections from the E18.0 cerebral cortex, which were electroporated with control (*n* = 3) or *Cdh6* KD vector (*n* = 3) (left) or control (CAG empty) (*n* = 3) or CAG‐*Cdh6*‐HA vectors (*n* = 3) (right) with CAG‐EGFP vectors at E14.0. (B) Western blot analysis of neocortical cells electroporated with CAG empty or CAG‐*Cdh6*‐HA vectors using antibodies against HA, Paxillin, pFAK, FAK, and GAPDH (a loading control) (*n* = 3). Sections in (A) were stained with DAPI (magenta). Scale bar: 50 μm in (A). KD, knockdown.

Our data suggest that CDH6 controls radial migration of neurons by activating integrin β1. Whether integrin β1 is involved in neuronal migration in the developing neocortex has long been debated. Previous studies using conventional KO, shRNA‐mediated KD, or neutralizing antibodies suggest that α3 and α5 are required for proper neuronal migration [26, 27]. However, genetic deletion of integrin β1, which is the sole binding partner for α3 and α5, did not affect the positioning of cortical pyramidal neurons when analyzed at juvenile and adult stages [25, 28]. More recently, specific genetic deletion of integrin β1 in postmitotic cortical neurons resulted in delayed radial migration and abnormal neuronal positioning when analyzed at pre‐ and perinatal stages, whereas no apparent abnormality was observed at the adult stage [31]. These results are consistent with the deletion of FAK and Paxillin, which act in the same pathway [39, 40], suggesting that the integrin β1/FAK/Paxillin complex contributes to proper neuronal migration. Although we did not detect any change in FAK activation and Paxillin protein expression levels in *Cdh6* overexpression and KD experiments (Fig. 7), our rescue experiment, in which integrin β1 overexpression partially but significantly rescued the migration defect caused by *Cdh6* KD, also supports the role of integrin β1 in radial neuronal migration. Some *Cdh6* KD neurons remained abnormally deep in the cerebral wall at P9 (Fig. 2G), while no apparent abnormality was observed in *integrin β1*‐deficient mice at P35 [31]. Although we cannot completely rule out the possibility that the migration defect is recovered between P9 and P35, the results suggest the possibility that some CDH6 functions independently of integrin β1, considering that active neuronal migration is not thought to occur after P9. Such functions, possibly through homophilic interaction of CDH6 or heterophilic binding to a binding partner other than integrin β1, may also contribute to proper radial migration and final positioning of cortical neurons.

How does CDH6 activate integrin β1? A previous report using colon cancer cells suggested a trans interaction between the RGD cadherin CDH17 and integrin, based on the observation that treatment with a soluble recombinant ectodomain of CDH17 caused integrin activation, but did not rule out a cis interaction [35]. Our observations that the active form of integrin β1 was decreased in *Cdh6* KD neurons, and that neuron‐specific expression of integrin β1 rescued the migration defect caused by *Cdh6* KD, suggest a cis interaction between CDH6 and integrin β1 on neurons. Consistent with this, a previous paper showed that inhibition of CDH6 in cultured neurons suppressed adhesion to laminin, one of the extracellular matrices to which integrin β1 can bind [12]. Several studies have shown that the cis binding partner controls integrin activation and cell adhesion in leukocytes. For example, cis interaction between integrin β2 and intracellular adhesion molecules (ICAMs) suppresses integrin activation and leukocyte adhesion [41]. Integrin β2 also interacts in cis with the IgG receptor FcγRIIA to reduce neutrophil recruitment under flow [42]. Thus, although we cannot exclude the possibility that the trans interaction between CDH6 and integrin β1, similar to the proposed trans interaction between RGD cadherin and integrin in other cells, also has functions in neurons, the cis interaction between CDH6 and integrin β1 may also modulate the conformation of integrin β1 on neurons. Cadherins undergo dynamic intracellular trafficking during various processes of tissue morphogenesis [43, 44]. During radial migration of cortical neurons, CDH2 trafficking is tightly regulated by multiple endocytic pathways mediated by small GTPases, including RAB5/RAB11 and ARF6/ARF4, and the adaptor protein DBNL [45, 46, 47, 48]. Thus, regulation of the cell surface levels of CDH6 might modulate the activation state of integrin β1 in migrating neurons. Several mechanisms are known to activate integrin β1 in migrating neurons, including Reelin/C3G/RAP1‐mediated inside‐out activation and paxillin‐mediated mechanisms [31, 32, 49]. However, it is not known whether these multiple mechanisms act completely independently or whether they work together during migration. Future studies are needed to clarify this issue.

In conclusion, we report that CDH6 is required for cortical excitatory neurons to migrate radially via control of integrin‐mediated cell motility. This study provides insight into the proper migration and positioning of excitatory neurons during the formation of the laminar structure of the cerebral cortex.

## Materials and methods

### Animals

Pregnant Slc:ICR mice were purchased from Japan SLC (Shizuoka, Japan). All the mice used in this study were housed in a standard specific pathogen‐free (SPF) facility and maintained on a 12‐h light/dark cycle (lights on at 8:30 AM, off at 8:30 PM) in an air‐conditioned room at 24 °C. All experimental procedures were approved by the Keio University Institutional Animal Care and Use Committee (No.: A2021‐030) and the National Institute of Neuroscience Institutional Animal Care and Use Committee in National Center of Neurology and Psychiatry (No.: 2023004) and were performed in accordance with the Institutional Guidelines on Animal Experimentation at Keio University and National Center of Neurology and Psychiatry, the Japanese Government Law Concerning the Protection and Control of Animals, and the Japanese Government Notification on Feeding and Safekeeping of Animals. The day of vaginal plug detection was considered as E0. Mice of both sexes were used in this study.

### Generation of *Cdh6*‐HA knock‐in mice

*Cdh6*‐HA knock‐in mice were generated by CRISPR/Cas9‐mediated genome editing, as described elsewhere (Hotta M, in preparation). Briefly, a synthesized single‐stranded DNA donor containing *Cdh6* genomic sequences without the stop codon and in‐frame two tandem copies of HA (Eurofins Genomics, Tokyo, Japan) was electroporated into B6C3F1/Slc (Japan SLC) fertilized eggs together with Cas9 protein (NEB, Ipswich, MA, USA) and chemically synthesized guide RNA (FASMAC, Kanagawa, Japan) matched around the *Cdh6* stop codon, and then, healthy eggs after the electroporation were then transferred into the oviduct of the pseudopregnant Slc:ICR mice (Japan SLC). Genomic DNAs extracted from the tails of F0 knock‐in heterozygous mice were then sequenced for the entire genome‐edited region. After crossing with B6C3F1/Slc, heterozygous progenies were intercrossed for several generations to maintain the sequence‐verified allele, and homozygous knock‐in pups were used for the HA immunostaining.

### Histological analysis

Embryos or neonates were placed on ice for anesthesia and perfusion fixed with 4% paraformaldehyde (PFA) in 0.1 m sodium phosphate buffer (pH 7.4). Brains were postfixed in the same fixative for 45 min on ice, replaced in a 25% sucrose solution with phosphate‐buffered saline (PBS), embedded in OCT compound (Sakura, Tokyo, Japan), and frozen in liquid nitrogen. The frozen sections were then cut into 20‐μm‐thick sections using a cryostat (CM3050 S; Leica, Wetzlar, Germany). For immunostaining, after rinsing in PBS, the sections were incubated in blocking solution (5% donkey or goat serum and 0.1%Triton X‐100 in PBS) for 30 min, then with primary antibodies diluted in blocking solution at 4 °C overnight, and then with species‐specific fluorescent secondary antibodies (1 : 1000; Thermo Fisher Scientific, Waltham, MA, USA, or 1 : 1000; Jackson ImmunoResearch Laboratories, West Grove, PA, USA) for 2 h at room temperature. The primary antibodies used in this study were as follows: CDH6 (sheep; R&D Systems, Minneapolis, MN, USA, AF2715, 1 : 200), HA (rat; Roche, Basel, Switzerland, 3F10, 1 : 500), Nestin (chick; Aves Labs, Davis, CA, USA, #NES, 1 : 400), DCX (rabbit; CST, Danvers, MA, USA, #4604, 1 : 500), RORB (mouse; Perseus Proteomics, Tokyo, Japan, PPN7927‐00, 1 : 400), CUX1 (rabbit; Proteintech, Rosemont, IL, USA, 11733‐1AP, 1 : 600), CD29 (clone 9EG7) (rat; BD, San Jose, CA, USA, 550531), Paxillin (rabbit; Abcam, Cambridge, MA, USA, ab32084, 1 : 200), pFAK (Y397) (rabbit, CST, 3283S, 1 : 1000). For control staining for CDH6 and HA, normal sheep IgG (ChromPure sheep IgG, whole molecule; Jackson ImmunoResearch Laboratories, cat# 013‐000‐003) and normal rat IgG (ChromPure rat IgG, whole molecule; Jackson ImmunoResearch Laboratories, cat# 102‐000‐003) were used, respectively. For 9EG7 immunostaining, biotin‐conjugated secondary antibody (1 : 1000; Jackson ImmunoResearch Laboratories), VECTASTAIN Elite ABC Kit (Vector, Newark, CA, USA) and TSA Plus Tetramethylrhodamine kit (PerkinElmer, Shelton, CT, USA) were used. DAPI (4,6‐di‐amidino‐2‐phenylindole, dihydrochloride; Invitrogen, Carlsbad, CA, USA) was used for nuclear staining. Fluorescence images were captured using a confocal laser scanning microscope (FV1000; Olympus, or TCS SP8; Leica). Images were processed using the adobe photoshop software and imagej2 version 2.9.0. To measure the apical dendrite angle of EGFP‐positive neurons located in the layer II/III (Fig. 2J), a perpendicular line was drawn from the pial surface to the center of the target cell body, from which another straight line was drawn to the first branch point of the apical dendrite. The angle between these two straight lines was measured using fiji software and defined as the apical dendrite angle (θ) as previously described [50].

### Plasmids

The pCAGGS‐EGFP, Tα1 expression vector, pTα1‐*integrin β1*, and pCAGGS‐*integrin β1* have been described previously [32, 51].

To construct a short *Cdh6* hairpin RNA (shRNA) expression vector, oligonucleotides targeting the *Cdh6* coding sequence (gacagcgttgctcaacatg) and its complementary sequence were inserted into the pSilencer 3.0‐H1 vector (Thermo Fisher Scientific). The pSilencer 3.0‐H1 vector was used as a negative control.

The HA‐tagged full‐length cDNA of *Cdh6* was amplified by RT‐PCR using cDNA from E17.5 ICR mouse brain as a template and cloned into the pBluescript KS by in‐Fusion ligation technology (Takara, Tokyo, Japan) (*Cdh6*‐WT‐HA/pBSKS). The following primers were used: forward primer (Cdh6‐F‐IF), ACGGTATCGATAAGCTTGATATGAGAACTTACCGGTACTTC, reverse primer (Cdh6‐R‐HA‐IF), CCGGGCTGCAGGAATTCGATTTAAGCGTAATCTGGAACATCGTATGGGTA GGAGTCTTTGTCACTGTCC (underlines are the sequence corresponding to the Cdh6 coding region).

The HA‐tagged form of *Cdh6* resistant to the *Cdh6* KD (*Cdh6* WT*‐HA) was generated with three point mutations by PCR using *Cdh6*‐HA/pBSKS as template (*Cdh6* WT*‐HA/pBSKS). The following primers were used: sense primer (Cdh6‐res‐Fw), AAGACAGCATTACTGAACATGGATCGAGAAAACAG; antisense primer (Cdh6‐res‐Rv), CATGTTCAGTAATGCTGTCTTGATGATACCTG (underlines are the points of the mutations).

The HA‐tagged resistant form of *Cdh6* to the *Cdh6* KD with a mutated RGD motif in which the sequence of RGD was changed to RAE (*Cdh6* RGD mut*‐HA) was generated with two point mutations by PCR using *Cdh6* WT*‐HA/pBSKS as the template (*Cdh6* RGD mut*‐HA/pBSKS). The following primers were used: sense primer (Cdh6‐RGD mut‐Fw), AGAGCAGAAGGATCACTTAAATATATCCTTTCAG; antisense primer (Cdh6‐RGD mut‐Rv), TTCTGCTCTATCCTGGTCTGAATGTAACTTG (underlines are the points of the mutations).

These HA‐tagged *Cdh6* wild‐type or mutant cDNAs were cloned into the pCAGGS1 or Tα1 vector (kind gift from Drs Sakakibara and Miyata, Nagoya University, Japan) to generate pCAGGS1‐*Cdh6* WT*‐HA, pCAGGS1‐*Cdh6* RGD mut*‐HA, pTα1‐*Cdh6* WT*‐HA, and pTα1‐*Cdh6* RGD mut*‐HA. All constructs were confirmed by DNA sequencing.

### *In utero* electroporation

Pregnant mice were anesthetized by intraperitoneal administration of an anesthetic mixture [75 ng·mL^−1^ medetomidine hydrochloride (Domitor; Nippon Zenyaku Kogyo, Fukushima, Japan), 400 ng·mL^−1^ midazolam (Sandoz, Tokyo, Japan), and 0.5 mg·mL^−1^ butorphanol (Vetorphale; Meiji Seika Pharma, Tokyo, Japan) in PBS], and their intrauterine embryos were surgically manipulated as described previously [52]. *In utero* electroporation to transfect vectors into the embryonic neocortex and hippocampus was performed as described previously [14, 53, 54, 55]. Briefly, approximately 1–2 μL of the plasmid solution containing 0.01% Fast Green solution was injected into the lateral ventricle of the intrauterine embryos; then, electronic pulses [31 V (E12) or 34 V (E14), 50 ms, 950‐ms intervals, four times] were applied using an electroporator (CUY‐21SC; NEPA GENE, Chiba, Japan) with a forceps‐type electrode (CUY650P5, NEPA GENE). Details of the plasmid DNA concentrations for electroporation are described in Table S1.

### Cell culture, transfection, and western blotting

Human embryonic kidney (HEK) 293T cells (RRID: CVCL_0063) were from Dr Katsuhiko Mikoshiba (RIKEN, Japan). HEK293T were cultured in a Dulbecco's modified Eagle medium (DMEM) (Thermo Fisher Scientific) supplemented with 10% fetal bovine serum (Hyclone; Cytiva, Marlborough, MA, USA) and 5% penicillin–streptomycin (Thermo Fisher Scientific) at 37 °C in an atmosphere of 5% CO_2_. All experiments were performed with mycoplasma‐free cells. HEK293T cells were transfected using PEI‐max (Polyscience, Warrington, PA, USA), cultured for 2 days, and homogenized in a lysis buffer [50 mm Tris–HCl, pH7.5, 10% glycerol, 1% Triton X‐100, 150 mm NaCl, 100 mm NaF, 1 mm Na_3_VO_4_, 10 mm EGTA, 2 μg·mL^−1^ aprotinin, 2 μg·mL^−1^ leupeptin, and 50 μm phenylmethylsulfonyl fluoride (PMSF)], and separated by sodium dodecyl sulfate‐polyacrylamide gel electrophoresis (SDS/PAGE) (4–20% acrylamide; Bio‐Rad, Hercules, CA, USA) and blotted onto a polyvinyldifluoride (PVDF) membrane (Bio‐Rad). The membranes were blocked in 5% BSA in PBST (0.05% Tween 20 in PBS) for 1 h and then probed with the primary antibodies in 1% skim milk in PBST for 1 h, followed by treatment with horseradish peroxidase‐conjugated secondary antibodies (Agilent Technologies, Santa Clara, CA, USA; Jackson ImmunoResearch) for 40 min at 37 °C and ECL Prime Western blotting detection reagent (GE Healthcare, Chicago, IL, USA). Signals were detected and measured using a cooled charge‐coupled device camera (LAS‐4000mini; GE Healthcare). The following primary antibodies were used in this study: CDH6 (sheep; R&D Systems, AF2715, 1 : 200), HA (rat; Roche, 11867423001, 1 : 1500), GAPDH (mouse; Santa Cruz Biotechnology, Dallas, TX, USA, sc‐32233, 1 : 1000), integrin β1 (rabbit; Abcam, ab183666, 1 : 1200), β‐Actin (mouse; Sigma‐Aldrich, MO, USA, A5441, 1 : 1000), Paxillin (rabbit; Abcam, ab32084, 1 : 1000), pFAK (Y397) (rabbit; CST, 3283S, 1 : 1500), and FAK (mouse; BD, 610088, 1 : 1000).

### *In vitro* electroporation

ICR mouse cortices at E15 were dissected in HBSS, and the cortical cells were dissociated with Papain (Nacalai Tesque, Kyoto, Japan). *In vitro* electroporation was performed using the dissociated cells at a density of 1–2 × 10^7^ cells per milliliter in Opti‐MEM medium (Thermo Fisher Scientific) with an electroporator (CUY‐21SC; NEPA GENE), under the following conditions: porting pulse: 275 V, 0.5 ms, 50‐ms intervals, two times, 10% decay rate; transfer pulse: 30 V, 50 ms, 50‐ms intervals, five times, 40% decay rate. After the electroporation, the cells were seeded onto 60‐mm dishes in Neurobasal medium containing 10% FBS and incubated at 37 °C in 5% CO_2_ for 2 h. Then, the medium was replaced with Neurobasal medium containing 3% B27 and 400 μm l‐glutamine, and the dishes were incubated at 37 °C in a humidified 5% CO_2_ atmosphere for 2 days. Cells were lysed and analyzed by western blotting.

### Integrin activation assay

The integrin activation assay was performed as previously described [32, 33] with some modifications. A 12‐well plate was coated with both poly‐l‐lysine (10 μg·mL^−1^; Sigma‐Aldrich). E17.0 embryonic cortices, into which control or *Cdh6* KD vector was introduced by IUE at E14.0, were dissected, incubated in warm PBS (37 °C) for 15 min, and mechanically dissociated by pipetting, followed by cell straining (BD). EGFP‐labeled cells were then collected by fluorescence activated cell sorting (FACS) using MoFlo XDP (Beckman Coulter, Brea, CA, USA). FACS sorted cells were plated on the coated dish and incubated at 37 °C for 40 min. After two washes with warm DMEM, 9EG7 antibody (2 μg·mL^−1^ in DMEM) was applied, followed by incubation at 37 °C for 15 min. After three washes with warm DMEM, cells were lysed in SDS sample buffer. Bound 9EG7 antibodies were detected with biotin‐conjugated donkey anti‐rat IgG (1 : 800; Jackson ImmunoResearch, 712‐065‐153) followed by HRP‐conjugated streptavidin (1 : 1000; Jackson ImmunoResearch, 016‐030‐084). Signals of activated integrin β1 and total integrin β1 were normalized to β‐Actin signal.

### Time‐lapse imaging

Time‐lapse imaging was performed as described previously with slight modifications [3, 14, 56]. Briefly, E17.0 embryonic cortices, in which control or *Cdh6* KD vector was introduced by IUE at E14.0, were prepared. Coronal brain slices (270 μm thick) from the middle 1/3 of the forebrain, mounted in a low melting temperature agarose, were sectioned using a vibratome (VT 1000S; Leica). The slices were placed on a Millicell‐CM membrane (pore size: 0.4 μm, Millipore, Billerica, MA, USA) on a glass bottom dish (AGC Techno Glass, Shizuoka, Japan) and cultured in Neurobasal medium (Thermo Fisher Scientific) containing 2% B27 (Thermo Fisher Scientific), 10% FBS, and 500 μm l‐glutamine (Thermo Fisher Scientific). The dishes were then mounted in a 40% O_2_ incubator chamber (Tokai Hit, Shizuoka, Japan) attached to a confocal microscope (SP8). Then, 5–10 optical Z‐section images were taken at the indicated intervals, and the focal planes were merged. The movement of each cell was analyzed using Manual tracking, an imagej2 plugin.

### Statistical analyses

The distribution of the EGFP‐labeled cells was analyzed on coronal sections at the level of the dorsal recess of the third ventricle of the embryonic brain at E18.0. The entire cortex was divided into 10 equally spaced bins. The distances from the top of the cortex to the nuclei of the migrating cells were measured using the imagej2 software. To analyze the distribution of the EGFP‐labeled cells at P9, the entire cortical wall was divided into three equal parts and defined as the superficial/middle/deep part. All data are expressed as mean ± SEM. For direct comparisons, the data were analyzed using an unpaired two‐tailed Student's *t*‐test because neither normality nor homogeneity of variances of the dataset was rejected by the chi‐squared test or the *F* test, respectively. For multiple comparisons, the data were analyzed by one‐way ANOVA with Tukey's *post hoc* test because neither normality nor homogeneity of variances of the dataset was rejected by the Shapiro–Wilk test or Levene's test, respectively. A *P* value less than 0.05 was considered significant. Outliers were tested by the Smirnov–Grubbs test at a level of significance of 0.05.

## Conflict of interest

The authors declare no conflict of interest.

## Author contributions

YH designed the experiments; acquired, analyzed, and interpreted the data, and prepared the manuscript. RS acquired and analyzed the data. TH, HS, MH, YUI, and TI acquired the data. KN designed the experiments, interpreted the data, and prepared the manuscript.

## Peer review

The peer review history for this article is available at https://www.webofscience.com/api/gateway/wos/peer‐review/10.1111/febs.70150.

## Supporting information


**Table S1.** Details of the plasmid DNA concentrations.

## Data Availability

All data supporting the findings of this study are available within this article and its Supporting Information.
