# Supplementary material for: Cadherin‐6 controls neuronal migration during mouse neocortical development via an integrin‐mediated pathway
Source: FEBS J. 2025 May 28;292(20):5382–97. doi: 10.1111/febs.70150 (PMC12524979; doi:10.1111/febs.70150)
Supplement: Supplementary file 1 — Table S1. Details of the plasmid DNA concentrations. [file FEBS-292-5382-s001.pdf]

Supporting Information

Cadherin-6 controls neuronal migration during mouse neocortical development via an integrin-mediated pathway

Yuki Hirota[1], Rikaho Saito[1], Takao Honda[1,2], Hitomi Sano[1, 3], Mayuko Hotta[4], Yukiko U. Inoue[4], Takayoshi Inoue[4], and Kazunori Nakajima

[1]Department of Anatomy, Keio University School of Medicine, 35 Shinanomachi, Shinjuku-ku, Tokyo 160-8582, Japan; [2]Laboratory of Molecular Biology, Department of Biofunctional Analysis, Gifu Pharmaceutical University, 1-25-4 Daigakunishi, Gifu, 501-1196, Japan; [3]Department of Information and Management, Tokyo Online University, 1-7-3 Nishi-Shinjuku, Shinjuku-ku, Tokyo 160-0023, Japan; [4]Department of Biochemistry and Cellular Biology, National Institute of Neuroscience, National Center of Neurology and Psychiatry, 4-1-1 Ogawahigashi, Kodaira, Tokyo, 187-8502, Japan.

This PDF file includes:
Table S1

Table S1. Details of the plasmid DNA concentrations.

Figure	Panel	Vector	Co	Vector	Co	Vector	Co	TC
1	H, I, J	pCAGGS-EGFP	0.8	-	-	-	-	0.8
2	B, D, E, G, I	pCAGGS-EGFP	0.8	pSilencer 3.0	2.5	-	-	3.3
		pCAGGS-EGFP	0.8	pSilencer Cdh6	2.5	-	-	3.3
	K	pCAGGS-EGFP	0.8	pSilencer 3.0	2.5	-	-	3.3
		pCAGGS-EGFP	0.8	pSilencer Cdh6	2.5	Tα1 (empty)	1.5	4.8
		pCAGGS-EGFP	0.8	pSilencer Cdh6	2.5	Tα1-*Cdh6* WT*	1.5	4.8
3	A	pCAGGS-EGFP	0.8	pSilencer 3.0	2.5	-	-	3.3
		pCAGGS-EGFP	0.8	pSilencer Cdh6	2.5	-	-	3.3
	C	pCAGGS-EGFP	0.8	pSilencer 3.0	2.5	-	-	3.3
		pCAGGS-EGFP	0.8	pSilencer Cdh6	2.5	-	-	3.3
4	A	pCAGGS-EGFP	0.8	pSilencer Cdh6	2.5	Tα1 (empty)	1.5	4.8
		pCAGGS-EGFP	0.8	pSilencer Cdh6	2.5	Tα1-*Cdh6* RGD mut*	1.5	4.8
5	A	pCAGGS-EGFP	0.8	pSilencer 3.0	2.5	-	-	3.3
		pCAGGS-EGFP	0.8	pSilencer Cdh6	2.5	-	-	3.3
		pCAGGS-EGFP	0.8	pSilencer Cdh6	2.5	CAG-*Cdh6* WT*	0.4	3.7
		pCAGGS-EGFP	0.8	pSilencer Cdh6	2.5	CAG-*Cdh6* RGD mut*	0.4	3.7
6	D, F, G	pCAGGS-EGFP	0.8	pSilencer Cdh6	2.5	Tα1 (empty)	1.5	4.8
		pCAGGS-EGFP	0.8	pSilencer Cdh6	2.5	Tα1-*integrin β1*	1.5	4.8
7	A	pCAGGS-EGFP	0.8	pSilencer 3.0	2.5	-	-	3.3
		pCAGGS-EGFP	0.8	pSilencer Cdh6	2.5	-	-	3.3
		pCAGGS-EGFP	0.8	CAG (empty)	0.8	-	-	1.6
		pCAGGS-EGFP	0.8	CAG-*Cdh6*	0.8	-	-	1.6

Co, concentration (mg/ml); TC, total concentration (mg/ml).
